# Pulmonary nocardiosis mimicking small cell lung cancer in ectopic ACTH syndrome associated with transformation of olfactory neuroblastoma: a case report

**DOI:** 10.1186/s12890-018-0710-9

**Published:** 2018-08-22

**Authors:** Keigo Kobayashi, Takanori Asakura, Makoto Ishii, Soichiro Ueda, Hidehiro Irie, Hiroyuki Ozawa, Kohei Saitoh, Isao Kurihara, Hiroshi Itoh, Tomoko Betsuyaku

**Affiliations:** 1 Division of Pulmonary Medicine, Department of Medicine, Keio University School of Medicine, 35 Shinanomachi, Shinjuku, Tokyo, 160-8582 Japan; 2 Department of Otorhinolaryngology, Head and Neck Surgery, Keio University School of Medicine, 35 Shinanomachi, Shinjuku, Tokyo, 160-8582, Japan; 3 Division of Endocrinology, Metabolism and Nephrology, Department of Medicine, Keio University School of Medicine, 35 Shinanomachi, Shinjuku, Tokyo, 160-8582, Japan

**Keywords:** Ectopic adrenocorticotropic hormone syndrome, Olfactory neuroblastoma, Pulmonary nocardiosis, Small cell lung cancer, Transformation

## Abstract

**Background:**

Pulmonary nocardiosis frequently develops as an opportunistic infection in cell-mediated immunosuppressive patients, and sometimes requires differentiation from pulmonary malignancy. Ectopic adrenocorticotropic hormone (ACTH) syndrome (EAS) is a neoplastic disorder which leads to impaired cell-mediated immunity, and is commonly associated with small cell lung cancer (SCLC). Because pulmonary infection and causative malignancy can appear as pulmonary lesions with EAS, differentiation of these diseases remains a critical issue for physicians.

**Case presentation:**

A 52-year-old woman with progressive lower limb paralysis and general fatigue was referred to us. She had been diagnosed with olfactory neuroblastoma (ONB) and treated with surgery and radiation therapy 10 years before the referral and had required stereotactic radiosurgery and chemotherapy 4 years later for a relapse of the ONB. On referral, she presented with Cushing’s syndrome with elevated cortisol and ACTH levels. Potassium supplement improved her symptoms; however, a month later, she was urgently hospitalized due to acute pleuritic chest pain on inspiration. Chest computed tomography revealed left lower lobular consolidations and a contralateral nodule in the right middle lobe. The clinical history and laboratory work-up suggested that her Cushing’s syndrome had most likely arisen from EAS. Additionally, the lungs were suspected as the ACTH source due to high levels of progastrin-releasing peptide and progressive pulmonary consolidation with a contralateral nodule, suggesting SCLC. However, histological examination from bronchoscopy revealed no evidence of malignancy, and *Nocardia cyriacigeorgica* was isolated from bronchoalveolar lavage fluid. Sulfamethoxazole/trimethoprim improved her pulmonary lesions. Somatostatin receptor scintigraphy revealed strong tracer uptake in the ONB lesions, indicating that the origin of the EAS was the olfactory tumor. However, histological examination of ONB specimens resected 10 years earlier showed no intracytoplasmic immunopositivity for ACTH.

**Conclusions:**

We highlight a rare case of pulmonary nocardiosis, which was associated with EAS mimicking SCLC, and was related to ONB transformation. Nocardiosis has to be considered even though anamnestic, clinical, and radiological aspects suggest the presence of metastasis. Additionally, physicians should carefully monitor patients with ONB for the development of Cushing’s symptoms because the tumor can transform into an ACTH-producing form, even after long-term follow-up.

## Background

Pulmonary nocardiosis is an uncommon gram-positive bacterial lung infection caused by aerobic actinomycetes of the genus *Nocardia*, which can be found in environmental sources such as soil, decomposing vegetation, other organic matter, and fresh and salt water [1]. It develops mainly as an opportunistic infection in patients with cell-mediated immunosuppressive conditions, including various malignancies and human immunodeficiency virus (HIV) infection, and those receiving long-term systemic steroids or immunosuppressive agents [1, 2]. In addition to nonspecific symptoms, pulmonary nocardiosis can present as various radiographic abnormalities. The most common findings include focal or multifocal consolidations and nodules, as well as cavitary lesions [1, 3]. It can be very difficult to clinically and radiographically differentiate pulmonary nocardiosis from other infections, such as fungal or mycobacterial infections, and malignancy [1, 4].

Ectopic adrenocorticotropic hormone (ACTH) syndrome (EAS) is a well-known paraneoplastic disorder, and accounts for about 10% of cases of all types of Cushing’s syndrome, and leads to impaired cell-mediated immunity by markedly raising cortisol levels [5]. The most prevalent tumors associated with EAS include small cell lung cancer (SCLC), neuroendocrine tumors, pheochromocytomas, and medullary carcinoma of the thyroid; it is said that the SCLC association is observed in over 50% of EAS patients [6]. Olfactory neuroblastoma (ONB), an indolent progressive tumor accounting for 3% of all intranasal tumors [7], can also cause EAS on rare occasions [8, 9].

Patients with EAS have a high prevalence of unusual pulmonary infections caused by opportunistic pathogens, including *Cryptococcus*, *Aspergillus*, *Pneumocystis*, and *Nocardia* [5, 10], which mimics lung malignancy as previously mentioned. Therefore, in patients with EAS of unknown origin, both pulmonary infection and causative malignancy of EAS can present as pulmonary lesions. Additionally, hypercortisolism in EAS may mask symptoms and signs of infection, leading to a delay in the diagnosis, which contributes to the high mortality rate [11]. Thus, differentiation of the pulmonary lesions with EAS remains a clinical issue for physicians.

Here, we report on a patient with ONB who was diagnosed with EAS of unknown origin and developed pulmonary lesions, which required differentiation from SCLC. We confirmed that the pulmonary lesions were caused by *Nocardia cyriacigeorgica* in EAS with ONB, which may have transformed to produce ACTH during the 10-year follow-up, as indicated by somatostatin receptor scintigraphy.

## Case presentation

**Fig. 1:**
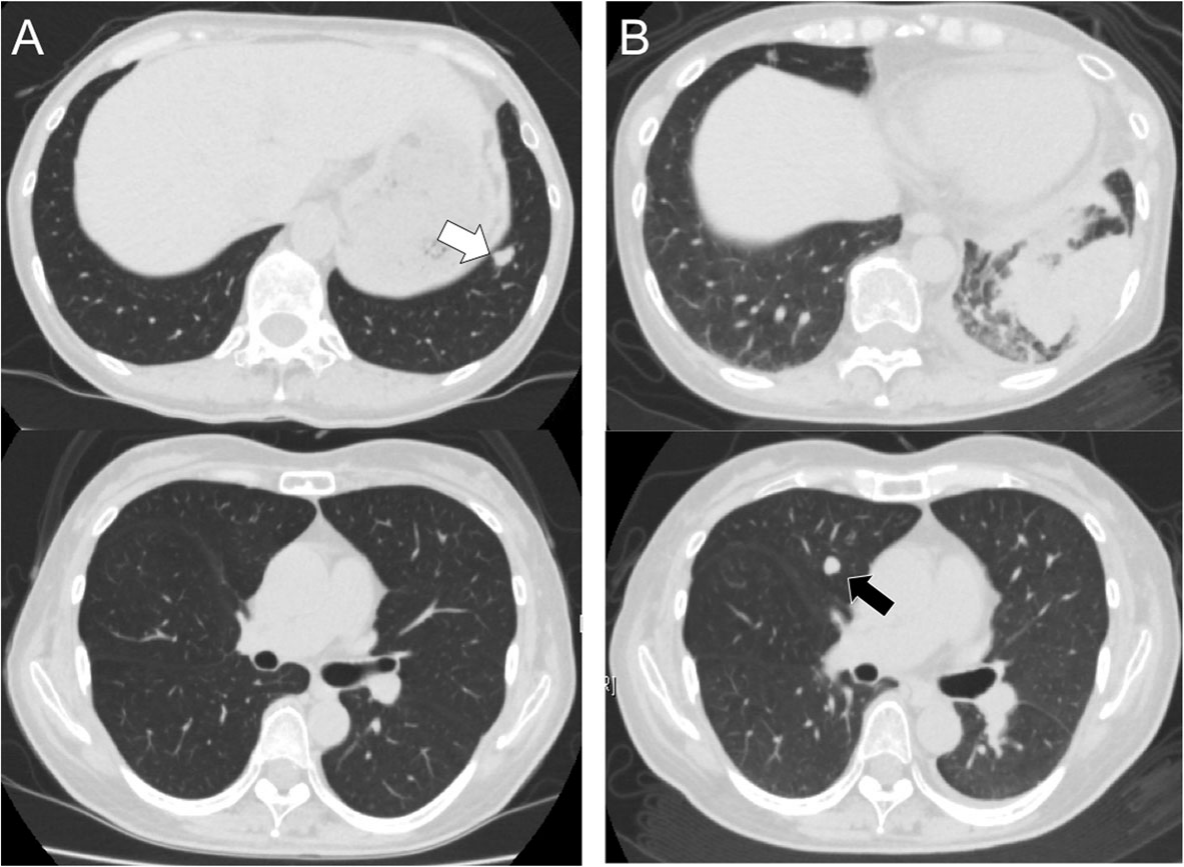
**a** Chest computed tomography (CT) performed 1 month before admission showed a pulmonary nodule in the left lower lobe (white arrow). **b** Chest CT on admission showed consolidations in the same lobe, and a new pulmonary nodule in the right middle lobe (black arrow)

A 52-year-old woman was referred to our hospital due to progressive lower limb paralysis and general fatigue. She had been diagnosed with ONB 10 years before the referral and had undergone skull base surgery and postoperative radiation therapy against ONB. Four years after her initial diagnosis, chemotherapy with paclitaxel, carboplatin, and cetuximab was administered after completion of CyberKnife (Accuray Inc., Sunnyvale, CA) treatment against a relapsed pterygopalatine fossa tumor. Although the tumor had gradually worsened after the treatment, the patient had been followed up without treatment because of its slow rate of growth. On referral, she had a Cushingoid appearance: moon face, central obesity, and thin skin with purpura. Laboratory examination revealed hypokalemia (2.4 mEq/L) and metabolic alkalosis. Elevated cortisol level (59.6 μg/dl, range 3.5–18.4), elevated ACTH level (469 pg/ml, range 7.2–63.3), and raised pro-gastrin releasing peptide level (ProGRP; 2110 pg/ml, range 0–81) were also detected. Chest and abdominal computed tomography (CT) revealed no abnormality except for a new pulmonary nodule in the left lower lobe (Fig. 1a). Head and neck magnetic resonance imaging (MRI) scans showed that there were no remarkable findings in the pituitary gland, and the ONB had slightly increased in size in the right nasal cavity and the right ethmoid sinus over 3 years. Thus, the history and laboratory data appeared to be consistent with EAS, however, an obvious source was not apparent. Potassium supplement improved her symptoms, and she was planned to be admitted for further work-up and management for EAS, at a later date.

One month after the referral, she was urgently hospitalized due to acute pleuritic chest pain on inspiration. She had no high fever (36.2 °C) or any other symptoms indicating respiratory infection. Chest CT revealed left lower lobular consolidations with pleural effusion and a new pulmonary nodule in the right middle lobe (Fig. 1b). Laboratory results revealed elevated levels of C-reactive protein (CRP: 12.77 mEq/L, range 0.00–0.35) and β-D-glucan (77.8 pg/ml, range 0–11), but were negative for *Aspergillus* antigen, *Aspergillus* precipitating antibody, and *Cryptococcus* antigen test. Her HIV screening was negative. Blood and sputum culture showed no remarkable findings. As evidence for Cushing’s syndrome, a raised cortisol level (72.3 μg/dl at 6 a.m., range 3.5–18.4) and ACTH level (466 pg/ml, 7.2–63.3) were also detected. The cortisol level was not suppressed by a low-dose dexamethasone suppression test (LDDST: 1 mg dexamethasone per day for 48 h): plasma cortisol level was 59.3 μg/dl and 24 h urine cortisol level was 439 μg/day after the LDDST. The history and laboratory work-up suggested that her Cushing’s syndrome was more likely arising from EAS. The lungs were suspected as the source of ACTH due to the high level of ProGRP and progressive pulmonary consolidation with a contralateral nodule, suggesting SCLC.

**Fig. 2:**
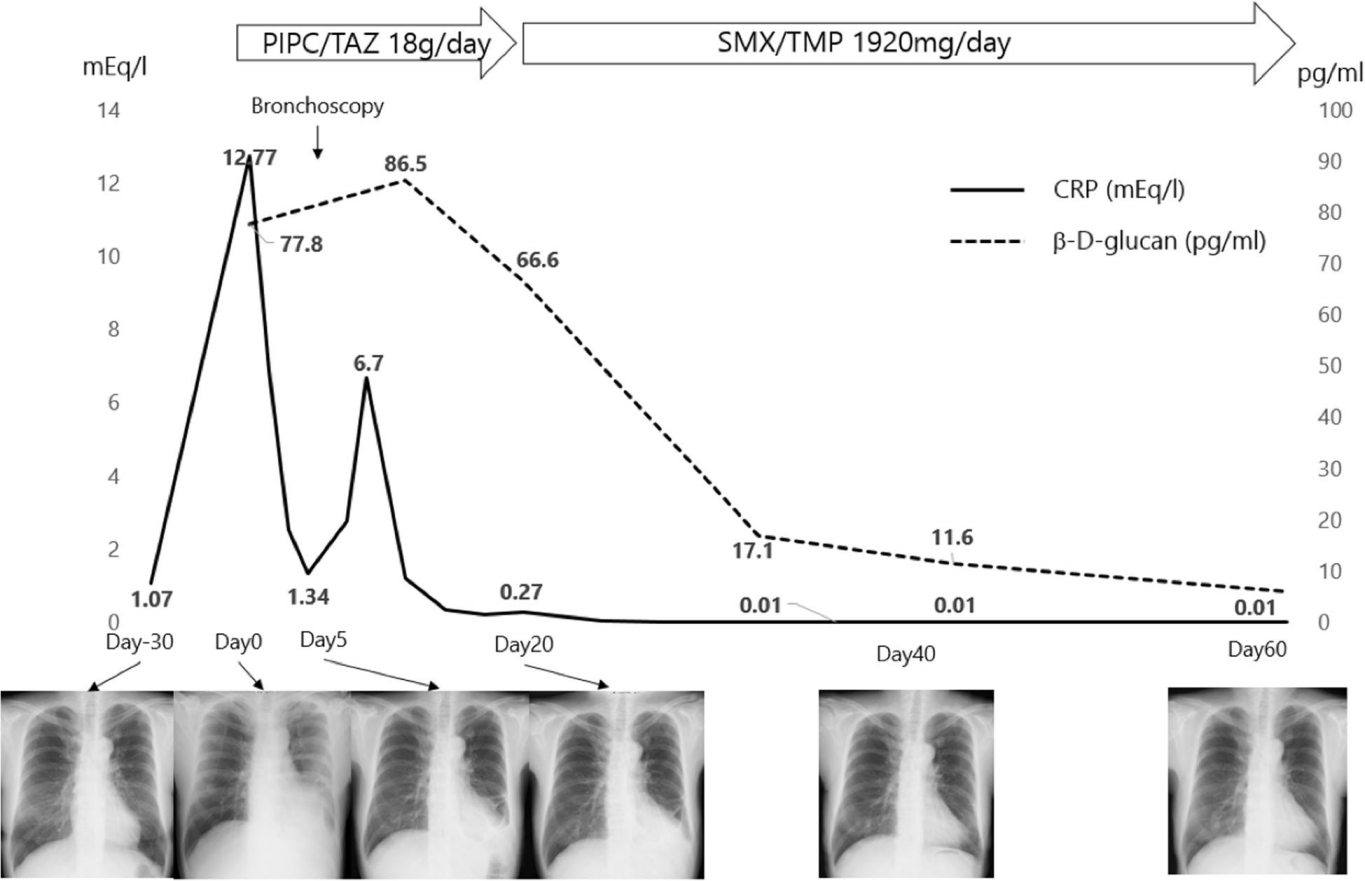
Clinical course of our case including the changes of serum CRP (solid line) and β-D-glucan (dotted line) levels is shown. Dates of the referral and admission are presented as day − 30 and day 0, respectively. Bronchoscopy was performed at day 5. PIPC/TAZ; piperacillin/tazobactum, SMX/TMP; sulfamethoxazole/trimethoprim, CRP; c-reactive protein

**Fig. 3:**
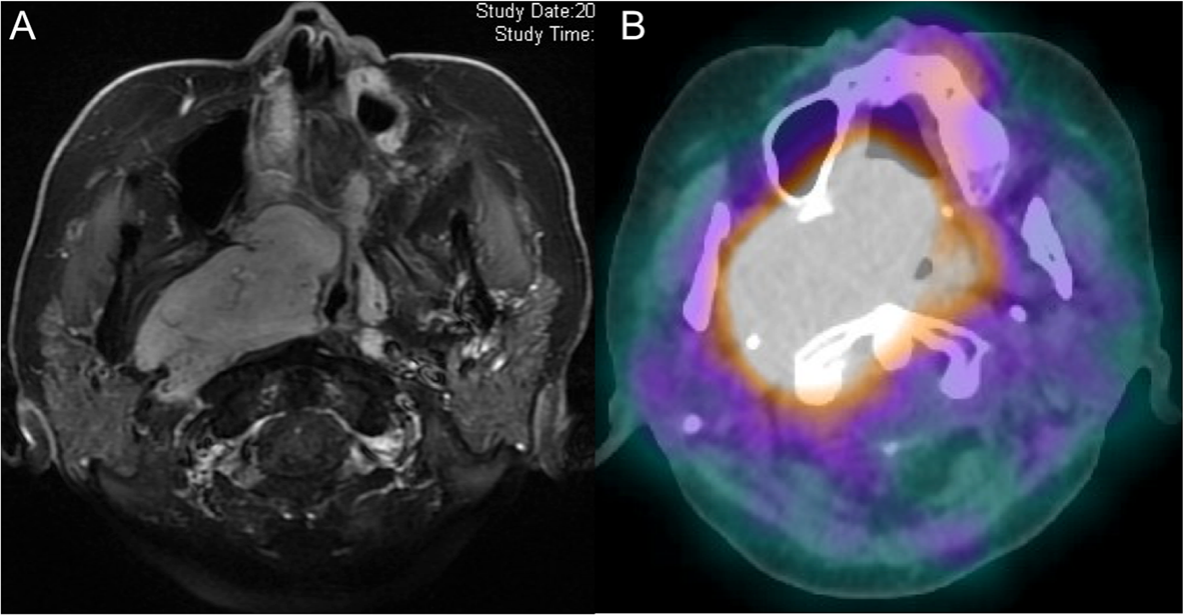
**a** T1-weighted contrast-enhanced magnetic resonance imaging of an axial image showed an enhancing mass in the right nasal cavity and ethmoid sinus. **b** Somatostatin receptor scintigraphy (Octreoscan) showed strong tracer uptake consistent with the tumor

Although treatment with piperacillin-tazobactam (18 g/day) for pneumonia improved her pleuritic chest pain after 5 days, the pulmonary consolidation did not resolve. Hence, we proceeded with bronchoscopy to differentiate SCLC from pulmonary infections. Histological examination of the transbronchial biopsy specimen and cytology of the bronchoalveolar lavage fluid (BALF) showed no evidence of malignancy or fungi. Two weeks later, the BALF culture was positive for *Nocardia spp.*, identified as *Nocardia cyriacigeorgica* by 16S ribosomal RNA gene sequencing. Table 1 shows the minimum inhibitory concentration of the indicated antibiotics against *N. cyriacigeorgica*. We used the Mueller-Hinton agar to culture *N. cyriacigeorgica* at 37 °C for 72 h. Susceptibility testing was performed according to Clinical and Laboratory Standards Institute document M24-A [12]. Oral sulfamethoxazole/trimethoprim 1920 mg (SMX 1600 mg/day, TMP 320 mg/day) improved her pulmonary lesions, as well as decreased the β-D-glucan level (Fig. 2). It was decided that the SMX/TMP would be continued for more than 1 year because she was an immunocompromised host [13]. Treatment with metyrapone (2.25 g/day) and mitotane (0.5 g/day) improved her plasma ACTH and cortisol levels. Scintigraphy performed using Octreoscan (Mallinckrodt, St Louis, MO) as an additional investigation for the origin of the EAS, revealed strong tracer uptake consistent with the ONB (Fig. 3); however, there was no uptake in the lung. Moreover, histological specimens of the ONB resected 10 years earlier showed no evidence of intracytoplasmic immunopositivity of ACTH. Therefore, this ONB may have transformed to produce ACTH over the 10-year clinical course.

**Table 1:** Antimicrobial Suscepibilities of an Isolated Strain of *N.cyriacigeorgica*

Agents	MIC (μg/ml)	Susceptible	Intermediate	Resistant
Amikacin	1	≤8	–	≥16
Amoxicillin / Clavulanate	> 32/16	≤8/4	16/8	≥32/16
Ceftriaxone	8	≤8	16–32	≥64
Ciprofloxacin	> 4	≤1	2	≥4
Imipenem	2	≤4	8	≥16
Linezolid	2	≤8	–	–
Minocycline	2	≤1	2–4	≥8
Sulfamethoxazole / Trimethoprim	19/1	≤38/2	–	≥76/4
Tobramycin	< 0.5	≤4	8	≥16
Cefotaxime	16	≤8	16–32	≥64
Cefepime	16	≤8	16	≥32
Doxycycline	2	≤1	2–4	≥8
Gentamicin	1	≤4	8	≥16
Ampicillin	> 8			
Clarithromycin	> 8	≤2	4	≥8
Erythromycin	> 2			

This table shows the minimum inhibitory concentration (MIC) of the indicated antibiotics against *N. cyriacigeorgica*. The specific susceptible breakpoint of Sulfamethoxazole / Trimethoprim (SMX/TMP) for Nocardia species was ≤2/38 and the specific resistance breakpoint was ≥4/76. The MICs of SMX/TMP for *N. cyriacigeorgica* were 1/19. Thus, the *N. cyriacigeorgica* was regarded as sensitive to SMX/TMP

## Discussion and conclusions

In the present case report, we highlight a rare case of pulmonary nocardiosis, which was associated with EAS mimicking SCLC, and was related to transformation of an ONB. Although there is still no clear cutoff value to distinguish between EAS and Cushing’s disease, plasma ACTH values of more than 200 pg/ml were reported in most EAS patients [14]. In this patient, matched clinical manifestations, higher plasma ACTH values, no remarkable findings in the hypothalamus and pituitary gland on brain MRI, and the confirmation with uptake consistent with the ONB in Octreoscan strongly supported a diagnosis of EAS, even though bilateral inferior petrosal sinus sampling, which is one of the confirmatory tests, was not performed because of its invasiveness and frequency of false-positives in ONB [15].

There are three important issues that made it difficult to differentiate pulmonary nocardiosis from SCLC in this case. First, the left pulmonary nodule was found in the first CT scan along with a high serum ProGRP level. Second, the chest CT on admission revealed a new pulmonary nodule in the right middle lobe, mimicking pulmonary metastasis. Third, the patient had no Cushingoid appearance over a 10-year period from the initial diagnosis of ONB, and the histological examination of resected specimens revealed no intracytoplasmic immunopositivity of ACTH.

*Nocardia* infection can mimic lung cancer in various situations: some reports revealed that cerebral or cutaneous nocardiosis mimics metastases of the lung [16]; pulmonary nocardiosis also mimics both primary and metastatic lung cancer because the *Nocardia* can gain access into the lung by direct inoculation or by hematogenous spread from the primary site [17]. Although a rapidly progressive radiological shadow (around 1 month) generally indicates an infective etiology rather than malignancy, SCLC could also cause rapid progression with poor prognosis. In our case, progressive pulmonary consolidation from a nodule and a new contralateral nodule during the follow-up suggested primary and metastatic lesions, respectively. Additionally, the presence of EAS and a high serum ProGRP level, one of the diagnostic markers for neuroendocrine tumors, such as SCLC, thyroid medullary carcinoma, and pancreatic endocrine tumor [6], made the diagnosis difficult.

Our patient also had a high level of β-D-glucan, which is a polysaccharide glucose polymer present in a broad range of commonly encountered fungal agents, including *Candida spp.*, *Aspergillus spp.*, and *Pneumocistis jirovecii*. Furthermore, a recent study reported a case of *Nocardia* infection with elevated serum β-D-glucan levels due to its possible cross-reactivity [18]. β-D-glucan may have been useful in the diagnosis of pulmonary infections in our situation; however, we could not exclude the possibility that these infections were complications of SCLC.

Our patient had Cushing’s syndrome due to EAS 10 years after the onset of the ONB, which may be as a result of the transformation that was confirmed by immunohistochemistry and Octreoscan. A recent study reviewed 18 cases of EAS with ONB: Cushing’s syndrome developed simultaneously with ONB diagnosis or its recurrence in nine cases; Cushing’s syndrome after diagnosis with ONB in five; and the location of the ACTH-secreting tumor was occult in four. The median times from ONB diagnosis to EAS development was 18 months [15], which is much shorter than in our case. In terms of confirming the source of ACTH production, Octreoscan is useful to diagnose ACTH-secreting tumors that have somatostatin receptors, which are expressed in 80% of EAS tumors [19]. Regarding immunohistochemical staining for ACTH, some tumors in Cushing’s syndrome have been shown to be negative due to their secretion of corticotropin-releasing hormone rather than ACTH [8]. Although investigation of the ACTH source was limited until the referral, we believe that the ONB underwent a transformation to start producing ACTH because Cushing’s syndrome was not evident until the referral, 10 years after the onset of the ONB.

In conclusion, we described a case of pulmonary nocardiosis mimicking SCLC in EAS, and associated with transformation of an ONB. In patients with EAS, physicians should consider both SCLC and pulmonary infection, including pulmonary nocardiosis, as differential diagnoses of pulmonary lesions. Additionally, physicians should carefully monitor patients with ONB for the development of Cushing’s symptoms because the tumor can transform to produce ACTH, even after a long-term follow-up.

## Abbreviations

ACTH: Adrenocorticotropic hormone
BALF: Bronchoalveolar lavage fluid
CRP: C-reactive protein
CT: Computed tomography
EAS: Ectopic adrenocorticotropic hormone syndrome
LDDST: Low-dose dexamethasone suppression test
MRI: Magnetic resonance imaging
ONB: Olfactory neuroblastoma
ProGRP: Pro-gastrin releasing peptide
SCLC: Small cell lung cancer
SMX/TMP: Sulfamethoxazole/trimethoprim
